# *Bordetella pertussis* Strain Lacking Pertactin and Pertussis Toxin

**DOI:** 10.3201/eid2202.151332

**Published:** 2016-02

**Authors:** Margaret M. Williams, Kathryn Sen, Michael R. Weigand, Tami H. Skoff, Victoria A. Cunningham, Tanya A. Halse, M. Lucia Tondella, CDC Pertussis Working Group

**Affiliations:** Centers for Disease Control and Prevention, Atlanta, Georgia, USA (M.M. Williams, M.R. Weigand, T.H. Skoff, M.L. Tondella);; New York State Department of Health, Albany, New York, USA (K.A. Sen);; Livingston County Health Department, Mt. Morris, New York, USA (V.A. Cunningham);; Wadsworth Center, Albany (T.A. Halse)

**Keywords:** Pertussis, *Bordetella pertussis*, pertussis toxin, pertactin

## Abstract

A *Bordetella pertussis* strain lacking 2 acellular vaccine immunogens, pertussis toxin and pertactin, was isolated from an unvaccinated infant in New York State in 2013. Comparison with a French strain that was pertussis toxin–deficient, pertactin wild-type showed that the strains carry the same 28-kb deletion in similar genomes.

Pertussis has resurged in the United States in recent decades; >48,000 cases were reported in 2012 (http://www.cdc.gov/pertussis/surv-reporting/cases-by-year.html). Suggested causes include improved surveillance and diagnostics, waning immune response to acellular vaccines introduced in the United States in the 1990s (DTaP [diphtheria, tetanus, and pertussis]; Tdap, [tetanus, diphtheria, and pertussis]), and changes to circulating *B. pertussis* strains, which led to a mismatch with vaccine strains (*1*). Components of acellular pertussis vaccines in the United States are pertactin (Prn), pertussis-toxin (Pt), filamentous hemagglutinin, and sometimes fimbrial proteins 2/3. Since 2010, multiple mutations have been documented in the Prn-encoding gene (*prn*), which have spread rapidly across the United States and other countries (*2*,*3*). Pt-deficient *Bordetella pertussis* isolates are rare, with 1 report from France (*4*). To our knowledge, *B. pertussis* that lacks Pt and an additional acellular vaccine immunogen has not been documented.

## The Case

Prodromal pertussis symptoms developed on March 4, 2013, in an 11-month-old white, non-Hispanic infant from New York State while the family was traveling outside the state. Cough reportedly began on March 14, 2013, and 12 days later (March 26) he was brought to his healthcare provider (HCP) with symptoms consistent with pertussis. Since the child’s birth, the diagnosing HCP had seen the child only once; no visits to other HCPs were known. Per parental report, the case-patient was experiencing paroxysmal cough, apnea, and posttussive vomiting. No thoracic radiograph was obtained. A 5-day course of oral azithromycin was prescribed; the parent reported that the infant received treatment for 3 consecutive days, beginning March 26, 2013. The infant was not reported to have any pertussis-associated complications (seizures, pneumonia, or encephalopathy) and had only light coughing as of April 11, 2013.

The infant was unvaccinated because the parents refused administration of all vaccines. Three siblings, ages 12, 10, and 8 years, lived with the infant and were undervaccinated; they had received 2, 1, and 3 doses, respectively, of pertussis-containing vaccines. No coughing illness was reported among the siblings. The mother reported that she received Tdap vaccine during her pregnancy with the case-patient, but receipt of vaccine could not be verified.

A nasopharyngeal swab specimen was collected from the infant on March 26, 2013, for testing at a commercial laboratory. The isolate was also forwarded to New York State’s public health laboratory, the Wadsworth Center, where it was found to be positive for *B. pertussis* by PCR targeting IS*481* and BP283 (*5*). Both laboratories yielded positive culture results for *B. pertussis*. No other testing was performed.

The Wadsworth Center forwarded the isolate, designated I979, to the Centers for Disease Control and Prevention (CDC; Atlanta, Georgia, USA) for confirmatory identification and molecular typing as part of the Enhanced Pertussis Surveillance program (*6*). PCR amplification of the gene encoding the first subunit of Pt (*ptxA*) was unsuccessful while the CDC multitarget real-time PCR diagnostic assay was performed (*7*). Amplification of the promoter region (*ptxP*) and *ptxA* was also unsuccessful during multilocus sequence typing targeting acellular vaccine component genes *ptxA*, *ptxP*, *prn*, and *fim3* (*8*).

Further characterization of I979 and French strain FR3749 (*4*) was undertaken by multilocus sequence typing, multilocus variable-number tandem-repeat analysis, pulsed-field gel electrophoresis (*9*), and whole-genome sequencing. Long sequencing reads were obtained with the Pacific Biosciences RS II (Menlo Park, CA, USA) at >120× coverage and assembled de novo into a single contig by using HGAP v3 and Quiver v1 (Pacific Biosciences). Assembly structure was confirmed with a genome optical map after restriction digestion with *Kpn*I (OpGen, Gaithersburg, MD, USA). The final sequence was polished with short reads obtained with Illumina MiSeq and CLC Genomics Workbench v7.5.1 (QIAGEN, Valencia, CA, USA) with >90× coverage. Completed genomes were submitted to the National Center for Biotechnology Information (http://www.ncbi.nlm.nih.gov/) with GenBank accession nos. CP010965 (FR3749) and CP010966 (I979). Basic genome metrics are listed in Table 1.

**Table 1:** Characterization of *Bordetella pertussis* strains I979 and FR3749 in comparison to strain Tohama I*

Strain	GenBank accession no.	Length, bp	No. IS*481*	*prn* type	Prn	Reference
FR3749	CP010965	4,079,396	249	2	+	This study
I979	CP010966	4,082,551	252	2	–	This study
Tohama I	NC_002929.2	4,086,189	238	1	+	(*10*)
*Prn, pertactin, +, positive; –, negative.						

**Figure 1:**
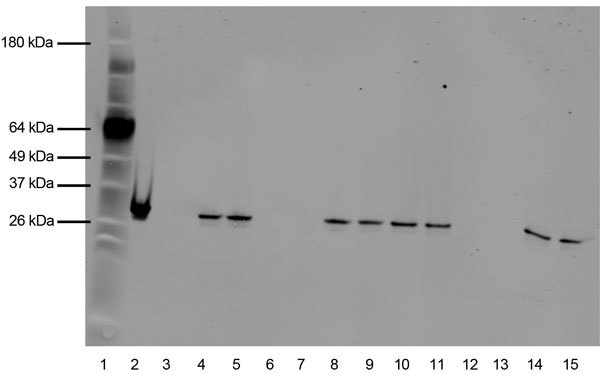
Western blot of pertussis toxin (Pt) expression in *Bordetella pertussis* Tohama I, I979, FR3749, and 3 additional recent isolates. All isolate lanes were loaded with 10-μg of protein, extracted after growth for 48 hours. Protein was transferred with the iBlot Dry Blotting system (Invitrogen, Carlsbad, CA, USA). The primary antibody consisted of 1b7 anti-PTX S1 monoclonal antibody at a concentration of 20 μg/mL diluted in 0.01 M PBS/Tween with 5% milk. The secondary antibody was a FITC-conjugated goat anti-mouse pAb from AbCam, diluted 1:1,000 with 0.01 M PBS/Tween with 5% milk. Lane 1, benchmark protein ladder, 6–180 kDa (Life Technologies, Grand Island, NY, USA); lane 2, Pt positive control, 2 μg; lane 3, empty; lanes 4 and 5, J024 (pertactin (Prn)+/Pt+); lanes 6 and 7, I979 (Prn-/Pt-); lanes 8 and 9, Tohama I (Prn+/Pt+); lanes 10 and 11, I978 (Prn-/Pt+); lanes 12 and 13, FR3749 (Prn+/Pt-); lanes 14 and 15, I974 (Prn-/Pt+).

Prn production was determined by ELISA (*2*,*11*). Pt production was examined through Western blot analysis of cultures grown in cyclodextrin-modified Stainer-Scholte liquid medium to optical density (OD) 600 nm = 0.1 (*12*). Proteins precipitated with trichloroacetic acid were washed, reduced, and separated by sodium dodecyl sulfate–polyacrylamide gel electrophoresis. Pt detection is described in Figure 1.

I979 and FR3749 share the same multilocus variable-number tandem-repeat analysis type 27 and *prn-2* genotype, the most common recent types (*3*,*13*). I979 and FR3749 are *fim3–1* and *fim3–2*, respectively. The *fim3* locus has fluctuated between these 2 alleles recently (*3*,*8*). Pulsed-field gel electrophoresis indicated that I979 displays profile CDC306, and FR3749 displays CDC046. I979 and FR3749 both lacked Pt production, as shown by Western blot (Figure 1). I979 also failed to produce Prn, whereas FR3749 was positive for Prn production by ELISA, within the range of negative (OD 0.3–0.6) and positive (OD 1.2–1.6) controls.

**Figure 2:**
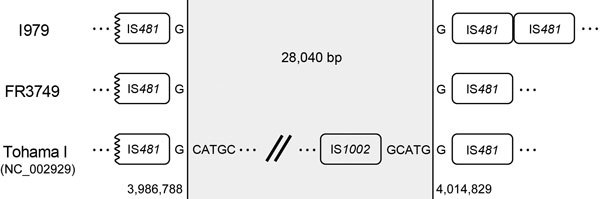
Map of the 28-kb region deleted in *Bordetella pertussis* strains I979 and FR3749, compared with vaccine strain Tohama I. Vertical lines indicate the deletion boundaries, at a nucleotide G of the IS*481* 6-base insertion site motif. The deleted region is flanked by a single IS*481* on each end in Tohama I and FR3749; I979 contains a second tandem IS*481* downstream of the deletion. In Tohama I, IS*1002* is located in the deletion region immediately upstream of IS*481,* and they share the 6-base motif.

Comparison of assembled I979 and FR3749 genomes with that of Tohama I (GenBank accession no. NC_002929.2) (*10*) indicated that the entire *ptx/ptl* operon is missing as the result of a putative deletion spanning 28,040 bp (Figure 2). Both genomes contain a conserved, truncated IS*481* immediately upstream of the deletion and a single IS*481* (FR3749) or 2 tandem IS*481* sequences (I979) immediately downstream (Figure 2). Within Tohama I, the region absent from I979 and FR3749 encodes 30 predicted genes bound by 2 NCATGN motifs, the target sequence for IS*481* insertion (Table 2). The insertion element IS*1002* is located within the 3′ end of this region, and shares a GCATGG motif with IS*481* immediately downstream. The 3′ deletion boundary is between IS*1002* and IS*481* (Figure 2). Whole-genome alignment, using progressiveMauve (*14*), of I979 and FR3749 with Tohama I revealed structural variation through genomic rearrangements and inversions. In particular, I979 and FR3749 genomes differ by a single, large inversion, the coordinates of which correspond to 2 conserved insertions of IS*481* in opposing orientations (Technical Appendix Figure). I979 and FR3749 differ by 31 single nucleotide polymorphisms, each differing from Tohama I by 204 and 173 single nucleotide polymorphisms, respectively. FR3749 contains wild-type *prn* at position 1613, whereas I979 *prn* contains an IS*481* insertion, the most common cause of Prn-deficiency, at position 1613 (*2*,*11*).

**Table 2:** Genes in *Bordetella pertussis* vaccine strain Tohama I deleted from *B. pertussis* I979 and FR3749 genomes, including the entire *ptx/ptl* operon*

Protein ID, GenBank accession no.	Gene	Product
NP_882281.1		Hypothetical protein
NP_882282.1	*ptxA*	Pertussis toxin subunit 1
NP_882283.1	*ptxB*	Pertussis toxin subunit 2
NP_882284.1	*ptxD*	Pertussis toxin subunit 4
NP_882285.1	*ptxE*	Pertussis toxin subunit 5
NP_882286.1	*ptxC*	Pertussis toxin subunit 3
NP_882287.1	*ptlA*	Type IV secretion system protein PtlA
NP_882288.1	*ptlB*	Type IV secretion system protein PtlB
NP_882289.1	*ptlC*	Type IV secretion system protein PtlC
NP_882290.1	*ptlD*	Type IV secretion system protein PtlD
NP_882291.1	*ptlI*	Type IV secretion system protein PtlI
NP_882292.1	*ptlE*	Type IV secretion system protein PtlE
NP_882293.1	*ptlF*	Type IV secretion system protein PtlF
NP_882294.1	*ptlG*	Type IV secretion system protein PtlG
NP_882295.1	*ptlH*	Type IV secretion system protein PtlH
		tRNA-Asn
NP_882296.1		Membrane protein
NP_882297.1		AraC family transcriptional regulator
NP_882298.1		Hypothetical protein
NP_882299.1		Membrane protein
NP_882300.1		Hypothetical protein
NP_882301.1		Peptide ABC transporter substrate binding protein
NP_882302.1		Transport system permease
NP_882303.1		Transport system permease
		Pseudogene
NP_882304.1	IS*481*	Transposase
NP_882305.1	*argJ*	Bifunctional ornithine acetyltransferase/N-acetylglutamate synthase
NP_882306.1		Hypothetical protein
NP_882307.1		Hypothetical protein
NP_882308.1	IS*1002*	Transposase

*ID, identification; *ptx*, pertussis toxin gene; *ptl*, pertussis toxin liberation gene.

## Conclusions

*B. pertussis* strain I979, identified in our study, is both Prn- and Pt-deficient. Loss of Pt in *B. pertussis* is a rare occurrence; only 2 isolates have been documented in 8 years. Both I979 and FR3749 were isolated from unvaccinated infants (11 months and 3 months old, respectively), who exhibited typical pertussis symptoms, although FR3749 had difficulty colonizing and multiplying in respiratory tracts of adult mice (*4*). *B. pertussis* isolates with deletions at other sites across the genome, including part or all of *prn*, were reported previously (*4*,*15*). During the past 5 years, US *B. pertussis* isolates have become nearly 100% Prn-deficient (*2*,*3*) (unpub. data), and Prn-deficient isolates have been obtained from vaccinated persons (*11*). The loss of Pt may represent a higher fitness cost to *B. pertussis* than the loss of Prn. In addition, the possibility that only the Pt-deficient isolates were recovered from patients who were co-infected with wild-type and mutant *B. pertussis* cannot be discarded. Further testing in models to understand the clinical relevance of Prn- and Pt-deficient strains in vaccinated and unvaccinated persons is warranted.

Although incidence of combined Pt- and Prn-deficiency in *B. pertussis* is rare, any increased mutation in these or other acellular vaccine immunogens may have serious implications for the efficacy of current vaccines. Global epidemiologic, culture-based, and molecular-based monitoring of *B. pertussis* is critical for understanding current trends of the disease it causes.

Technical Appendix Genomic rearrangement in *Bordetella pertussis* strains I979 and FR3749, compared with vaccine strain Tohama I.
