# Supplementary material for: Bordetella pertussis Strain Lacking Pertactin and Pertussis Toxin
Source: Emerg Infect Dis. 2016 Feb;22(2):319–22. doi: 10.3201/eid2202.151332 (PMC4734536; doi:10.3201/eid2202.151332)
Supplement: Technical Appendix — Genomic rearrangement in Bordetella pertussis strains I979 and FR3749, compared with vaccine strain Tohama I. [file 15-1332-Techapp-s1.pdf]

Article DOI: http://dx.doi.org/10.3201/eid2202.151332

Bordetella pertussis Lacking Pertactin and Pertussis Toxin

Technical Appendix

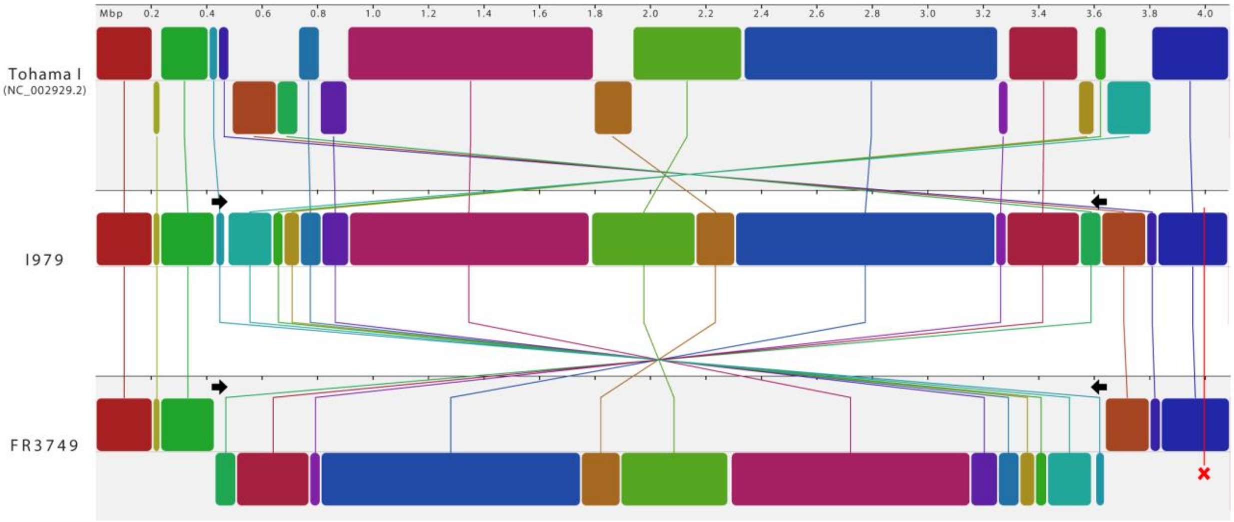

Technical Appendix Figure. Genomic rearrangement in *Bordetella pertussis* strains I979 and FR3749 compared with vaccine strain Tohama I, aligned in progressiveMauve (http://darlinglab.org/mauve/user-guide/progressivemauve.html). I979 and FR3749 differ by 1 large rearrangement. Location of the 28-kb deletion that includes the *ptx/ptl* operon is marked by a red vertical line.
